# Supplementary material for: Molecular Characteristics and Antimicrobial Resistance of Salmonella enterica Serovar Schwarzengrund from Chicken Meat in Japan
Source: Antibiotics (Basel). 2021 Nov 1;10(11):1336. doi: 10.3390/antibiotics10111336 (PMC8615118; doi:10.3390/antibiotics10111336)
Supplement: Supplementary file 1 [file antibiotics-10-01336-s001.zip › antibiotics-1443117-supplementary.pdf]

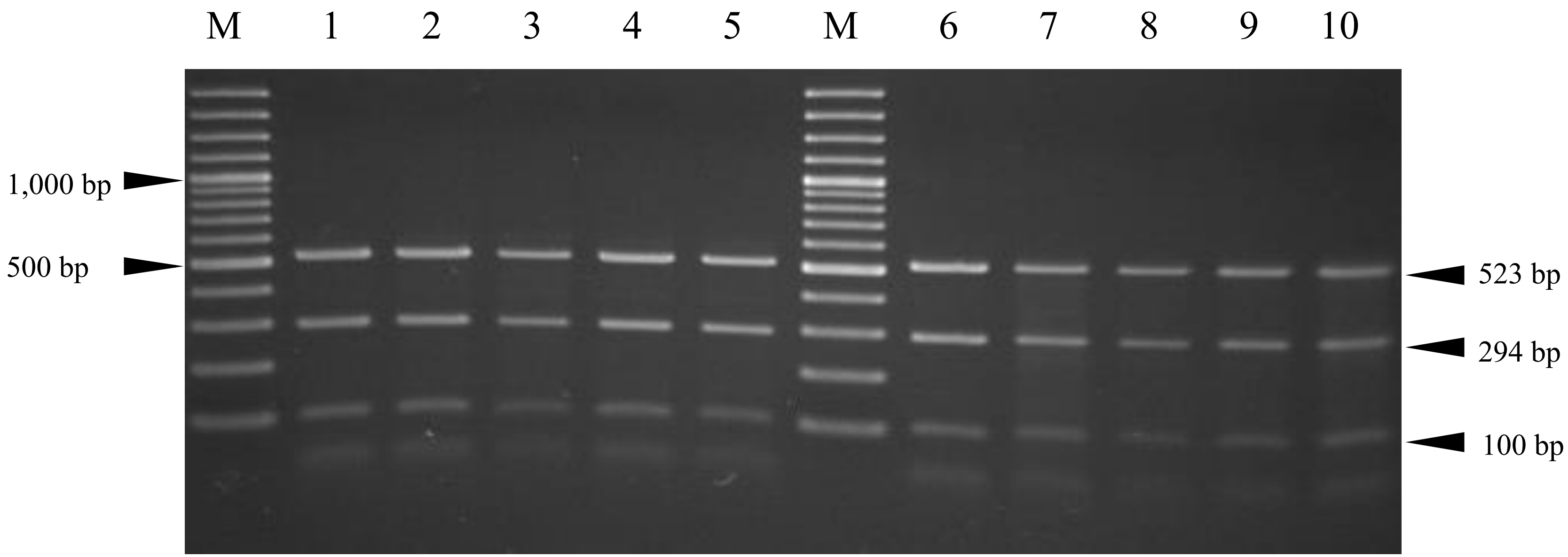

Figure S1, Agarose gel electrophoresis of Taq I-digested class 1 integron for *Salmonella enterica* serovar Schwarzengrund. Lanes: M, Gene Ruler 100 bp Plus Ladder (Thermo Fisher Scientific K.K., Tokyo); 1, Sal_G1* (2017, *aac(6')-Iaa-aadA1-tetA-sul1-dfrA14-aphA1*); 2, Sal_T1 (2008, *aac(6')-Iaa-aadA1-tetA-sul1-dfrA14-aphA1*); 3, Sal_T5 (2008, *aac(6')-Iaa-aadA1-tetA-sul1-dfrA14-aphA1*); 4, Sal_T15 (2008, *aac(6')-Iaa-aadA1-tetA-sul1-dfrA14*); 5, Sal_T22 (2008, *aac(6')-Iaa-aadA1-tetA-sul1-dfrA14*); 6, Sal_G1* (2017, *aac(6')-Iaa-aadA1-tetA-sul1-dfrA14-aphA1*); 7, Sal_17 (2015, *aac(6')-Iaa-aadA1-tetA-sul1-aphA1*); 8, Sal_100 (2016, *aac(6')-Iaa-aadA1-tetA-sul1*); 9, Sal_112 (2016, *aac(6')-Iaa-aadA1-tetA-sul1-dfrA14-aphA1*); 10, Sal_152 (2016, *aac(6')-Iaa-aadA1-tetA-sul1-dfrA14-aphA1*). Taq I-digested PCR products of class 1 integron were resolved in 2% agarose through electrophoresis. Isolation year and the detected antimicrobial resistance genes for each isolate are shown in parentheses.

* The nucleotide sequence of Class 1 integron of Sal_G1 was determined via DNA sequence analysis.

All nine isolates shown in this figure were identified as sequence type 241 through multilocus sequence typing.

Table S1. Antimicrobial resistance genes determined by ResFinder

Antimicrobial resistance gene	Sal_167[(a)]	Sal_249	Sal_266	Sal_278	Sal_291
aac(6')-Iaa	438/438[(b)] NC_003197[(c)]	438/438 NC_003197	438/438 NC_003197	438/438 NC_003197	438/438 NC_003197
ant(3'')-Ia (*aadA1*)	972/972 X02340	972/972 X02340	ND	972/972 X02340	ND
aph(3'')-Ib (*strA*)	ND	ND	ND	ND	804/804 AF321551
aph(6)-Id (*strB*)	ND	ND	ND	ND	837/837 M28829
aph(3')-Ia (*aphA1*)	ND	816/816 X62115	816/816 X62115	816/816 X62115	816/816 X62115
tetA	1241/1275 AF534183	1200/1200 AJ517790	ND	1240/1275 AF534183	ND
sul1	839/840 U12338	840/840 U12338	ND	837/840 U12338	ND
sul2	ND	ND	ND	ND	816/816 AY034138
dfrA14	474/474 AF393510	474/474 AF393510	ND	474/474 AF393510	474/474 AF393510

(a) Isolate code; (b) Alignment Length/Gene Length; (c) Accsession no.

ND, not determined

Coverage, 97.25-100

Table S2. Sequence types obtained by multilocus sequence typing for representative *Salmonella enterica* serovar Schwarzengrund isolates from retail chicken meat in Japan

	Year	Isolate no.	Antimicrobial resistance gene	MLST analysis		
				ST		Allelic profile
West Japan	2008	Sal_T1	*aac(6')-Iaa-aadA1-tetA-sul1-dfrA14-aphA1*	241		43-47-49-16-41-15-3
		Sal_T5	*aac(6')-Iaa-aadA1-tetA-sul1-dfrA14-aphA1*	241		43-47-49-16-41-15-3
		Sal_T15	*aac(6')-Iaa-aadA1-tetA-sul1-dfrA14*	241		43-47-49-16-41-15-3
		Sal_T22	*aac(6')-Iaa-aadA1-tetA-sul1-dfrA14*	241		43-47-49-16-41-15-3
		Sal_T29	*aac(6')-Iaa-aadA1-tetA-sul1-dfrA14*	241		43-47-49-16-41-15-3
		Sal_T30	*aac(6')-Iaa-aadA1-tetA-sul1-aphA1*	241		43-47-49-16-41-15-3
	2016	Sal_247	*aac(6')-Iaa-aadA1-tetA-sul1-dfrA14-aphA1*	241		43-47-49-16-41-15-3
	2017	Sal_276	*aac(6')-Iaa-aadA1-tetA-sul1-dfrA14-aphA1*	241		43-47-49-16-41-15-3
		Sal_G4	*aac(6')-Iaa-dfrA14-aphA1*	241		43-47-49-16-41-15-3
East Japan	2015	Sal_17	*aac(6')-Iaa-aadA1-tetA-sul1-aphA1*	241		43-47-49-16-41-15-3
	2016	Sal_100	*aac(6')-Iaa-aadA1-tetA-sul1*	241		43-47-49-16-41-15-3
		Sal_112	*aac(6')-Iaa-aadA1-tetA-sul1-dfrA14-aphA1*	241		43-47-49-16-41-15-3
		Sal_145	*aac(6')-Iaa-aphA1*	241		43-47-49-16-41-15-3
		Sal_152	*aac(6')-Iaa-aadA1-tetA-sul1-dfrA14-aphA1*	241		43-47-49-16-41-15-3
		Sal_167	*aac(6')-Iaa-aadA1-tetA-sul1-dfrA14*	241	*	43-47-49-16-41-15-3
		Sal_249	*aac(6')-Iaa-aadA1-tetA-sul1-dfrA14-aphA1*	241	*	43-47-49-16-41-15-3
	2017	Sal_270	*aac(6')-Iaa-strA/B-sul2-dfrA14-aphA1*	241		43-47-49-16-41-15-3
		Sal_G27	*aac(6')-Iaa-aphA1*	241		43-47-49-16-41-15-3
		Sal_266	*aac(6')-Iaa-aphA1*	241	*	43-47-49-16-41-15-3
		Sal_278	*aac(6')-Iaa-aadA1-tetA-sul1-dfrA14-aphA1*	241	*	43-47-49-16-41-15-3
	2019	Sal_301	*aac(6')-Iaa*	241		43-47-49-16-41-15-3
		Sal_291	*aac(6')-Iaa-strA/B-sul2-dfrA14-aphA1*	241	*	43-47-49-16-41-15-3
Domestic**	2015	Sal_63	*aac(6')-Iaa-aadA1-tetA-sul1-dfrA14-aphA1*	241		43-47-49-16-41-15-3
	2017	Sal_G1	*aac(6')-Iaa-aadA1-tetA-sul1-dfrA14-aphA1*	241		43-47-49-16-41-15-3
		Sal_G31	*aac(6')-Iaa-aphA1*	241		43-47-49-16-41-15-3

* ST was determined by WGS.

**The product district could not be identified

MLST, multilocus sequence typing; ST, sequence type.

Table S3. Sequence types of *Salmonella enterica* serovar Schwarzengrund obtained by multilocus sequence typing analysis and their geographical distribution.

MLST analysis		No. of strains	Distribution					
ST	Allelic profile		United States	United Kingdom	Taiwan	Brazil	Canada	Other countries
96 [(c)]	43-47-49-49-41-15-3	1,609	1,383	129	16	16	12	53 [(a)]
241 [(c)]	43-47-49-**<u>16</u>**-41-15-3	30	3	16	1	0	0	10 [(b)]
322 [(c)]	43-47-49-49-41-15-**<u>114</u>**	12	0	1	5	0	0	6
5010 [(c)]	43-47-49-49-41-15-**<u>908</u>**	3	3	0	0	0	0	0
2114 [(c)]	43-47-49-**<u>149</u>**-41-15-**<u>4</u>**	2	0	1	0	0	0	1
7288 [(c)]	43-47-49-49-**<u>1147</u>**-15-3	2	0	0	2	0	0	0
848 [(c)]	43-47-**<u>25</u>**-49-41-15-3	1	0	0	0	0	0	1
5405 [(c)]	43-**<u>810</u>**-49-49-41-15-3	1	1	0	0	0	0	0
6132 [(c)]	43-47-49-49-41-15-**<u>1035</u>**	1	1	0	0	0	0	0
7884 [(c)]	43-47-49-49-41-15-**<u>1257</u>**	1	1	0	0	0	0	0
2250 [(d)]	**<u>516</u>**-**<u>13</u>**-**<u>401</u>**-**<u>131</u>**-**<u>522</u>**-**<u>2</u>**-**<u>531</u>**	14	2	12	0	0	0	0
5815 [(d)]	**<u>516</u>**-**<u>13</u>**-**<u>401</u>**-**<u>131</u>**-**<u>522</u>**-**<u>2</u>**-**<u>989</u>**	1	0	1	0	0	0	0
213 [(e)]	**<u>10</u>**-**<u>7</u>**-**<u>12</u>**-**<u>9</u>**-**<u>70</u>**-**<u>9</u>**-**<u>2</u>**	2	0	0	0	0	0	2
19 [(e)]	**<u>10</u>**-**<u>7</u>**-**<u>12</u>**-**<u>9</u>**-**<u>5</u>**-**<u>9</u>**-**<u>2</u>**	1	1	0	0	0	0	0
2488	**<u>516</u>**-**<u>4</u>**-**<u>15</u>**-**<u>131</u>**-**<u>522</u>**-**<u>518</u>**-**<u>531</u>**	3	0	2	0	0	0	1
64	**<u>10</u>**-**<u>14</u>**-**<u>15</u>**-**<u>31</u>**-**<u>25</u>**-**<u>20</u>**-**<u>33</u>**	2	1	0	0	0	0	1
152	**<u>62</u>**-**<u>53</u>**-**<u>54</u>**-**<u>60</u>**-**<u>5</u>**-**<u>53</u>**-**<u>54</u>**	2	2	0	0	0	0	0
239	**<u>50</u>**-**<u>80</u>**-**<u>25</u>**-**<u>85</u>**-**<u>40</u>**-**<u>58</u>**-**<u>4</u>**	2	0	2	0	0	0	0
2885	**<u>480</u>**-**<u>450</u>**-**<u>8</u>**-**<u>204</u>**-**<u>491</u>**-**<u>103</u>**-**<u>303</u>**	2	0	0	0	0	0	2
11	**<u>5</u>**-**<u>2</u>**-**<u>3</u>**-**<u>7</u>**-**<u>6</u>**-**<u>6</u>**-**<u>11</u>**	1	1	0	0	0	0	0
13	**<u>3</u>**-**<u>3</u>**-**<u>7</u>**-**<u>4</u>**-**<u>3</u>**-**<u>3</u>**-**<u>7</u>**	1	1	0	0	0	0	0
14	**<u>7</u>**-**<u>6</u>**-**<u>8</u>**-**<u>8</u>**-**<u>7</u>**-**<u>8</u>**-**<u>13</u>**	1	1	0	0	0	0	0
45	**<u>10</u>**-**<u>7</u>**-**<u>21</u>**-**<u>14</u>**-**<u>15</u>**-**<u>12</u>**-**<u>12</u>**	1	0	0	0	1	0	0
226	**<u>84</u>**-**<u>76</u>**-**<u>38</u>**-**<u>16</u>**-**<u>12</u>**-**<u>13</u>**-**<u>4</u>**	1	1	0	0	0	0	0
279	**<u>62</u>**-**<u>95</u>**-**<u>54</u>**-**<u>96</u>**-**<u>100</u>**-**<u>9</u>**-**<u>100</u>**	1	0	1	0	0	0	0
319	**<u>118</u>**-**<u>107</u>**-**<u>8</u>**-**<u>51</u>**-**<u>2</u>**-**<u>117</u>**-**<u>16</u>**	1	1	0	0	0	0	0

404	46-122-3-18-6-138-133	1	1	0	0	0	0	0
419	13-11-25-72-10-23-23	1	1	0	0	0	0	0
548	13-11-25-197-12-71-4	1	0	0	0	1	0	0
2045	16-16-26-18-478-12-18	1	0	0	0	0	0	1
2046	47-64-108-96-491-455-303	1	0	1	0	0	0	0
3752	111-11-577-74-388-71-4	1	0	1	0	0	0	0
5226	346-64-25-86-76-22-41	1	0	1	0	0	0	0
5927	22-11-25-21-224-892-1007	1	0	1	0	0	0	0
6221	39-86-108-204-76-42-86	1	0	0	0	0	0	1
Total		1707	1405	169	24	18	12	79

These data were obatined from the *Salmonella* Genome Databases [22] and EnteroBase [23].

Allele types that differ from ST96 are underlined and in bold.

MLST, multilocus sequence typing; ST, sequence type.

(a) Ireland (7), Austria (6), Germany (4), Trinidad and Tobago (4), Scotland (3), Vietnam (3), Denmark (2), Ecuador (2), Northern Ireland (2), Thailand (2), South Africa (2), Indonesia (1), Italy (1), Netherlands (1), Poland (1), Puerto Rico (1), Tunisia (1), Unknown (10).

(b) India (4), Ireland (1), Northern Ireland (1), Poland (1), Zimbabwe (1), Unknown (2).

(c), (d), (e) Each group consists of related STs that differed in one or two loci only.

Table S4. Number of bases different among the seven genes from ST96.

ST	Allelic profile	*aroC*	*dnaN*	*hemD*	*hisD*	*purE*	*sucA*	*thrA*	total
96	43-47-49-49-41-15-3	-	-	-	-	-	-	-	-
241	43-47-49-**16**-41-15-3	0	0	0	7	0	0	0	7
322	43-47-49-49-41-15-**114**	0	0	0	0	0	0	1	1
5010	43-47-49-49-41-15-**908**	0	0	0	0	0	0	1	1
2114	43-47-49-**149**-41-15-**4**	0	0	0	1	0	0	3	4
7288	43-47-49-49-**1147**-15-3	0	0	0	0	1	0	0	1
848	43-47-**25**-49-41-15-3	0	0	1	0	0	0	0	1
5405	43-**810**-49-49-41-15-3	0	1	0	0	0	0	0	1
6132	43-47-49-49-41-15-**1035**	0	0	0	0	0	0	1	1
7884	43-47-49-49-41-15-**1257**	0	0	0	0	0	0	1	1
2250	**516**-**13**-**401**-**131**-**522**-**2**-**531**	4	7	2	6	7	8	11	45
5815	**516**-**13**-**401**-**131**-**522**-**2**-**989**	4	7	2	6	7	8	12	46
213	**10**-**7**-**12**-**9**-**70**-**9**-**2**	3	8	6	9	8	6	6	46
19	**10**-**7**-**12**-**9**-**5**-**9**-**2**	3	8	6	9	7	6	6	45
2488	**516**-**4**-**15**-**131**-**522**-**518**-**531**	4	8	5	6	7	8	11	49
64	**10**-**14**-**15**-**31**-**25**-**20**-**33**	3	8	5	9	8	7	4	44
152	**62**-**53**-**54**-**60**-**5**-**53**-**54**	4	8	6	9	7	9	5	48
239	**50**-**80**-**25**-**85**-**40**-**58**-**4**	1	13	1	7	4	4	3	33
2885	**480**-**450**-**8**-**204**-**491**-**103**-**303**	6	11	3	6	6	7	7	46
11	**5**-**2**-**3**-**7**-**6**-**6**-**11**	3	9	3	8	7	5	6	41
13	**3**-**3**-**7**-**4**-**3**-**3**-**7**	5	8	4	5	8	8	6	44
14	**7**-**6**-**8**-**8**-**7**-**8**-**13**	3	9	3	6	8	4	11	44
45	**10**-**7**-**21**-**14**-**15**-**12**-**12**	3	8	5	8	4	9	10	47
226	**84**-**76**-**38**-**16**-**12**-**13**-**4**	3	3	5	7	2	7	3	30
279	**62**-**95**-**54**-**96**-**100**-**9**-**100**	4	11	6	6	9	6	5	47
319	**118**-**107**-**8**-**51**-**2**-**117**-**16**	4	5	3	6	7	6	8	39
404	**46**-**122**-**3**-**18**-**6**-**138**-**133**	2	9	3	8	7	13	4	46
419	**13**-**11**-**25**-**72**-**10**-**23**-**23**	2	8	1	4	14	6	4	39
548	**13**-**11**-**25**-**197**-**12**-**71**-**4**	2	8	1	8	2	7	3	31
2045	**16**-**16**-**26**-**18**-**478**-**12**-**18**	4	11	4	8	9	9	8	53

2046	**<u>47</u>-<u>64</u>-<u>108</u>-<u>96</u>-<u>491</u>-<u>455</u>-<u>303</u>**	3	10	4	6	6	5	7	41
3752	**<u>111</u>-<u>11</u>-<u>577</u>-<u>74</u>-<u>388</u>-<u>71</u>-<u>4</u>**	4	8	2	5	4	7	3	33
5226	**<u>346</u>-<u>64</u>-<u>25</u>-<u>86</u>-<u>76</u>-<u>22</u>-<u>41</u>**	4	10	1	5	6	10	4	40
5927	**<u>22</u>-<u>11</u>-<u>25</u>-<u>21</u>-<u>224</u>-<u>892</u>-<u>1007</u>**	3	8	1	2	13	3	3	33
6221	**<u>39</u>-<u>86</u>-<u>108</u>-<u>204</u>-<u>76</u>-<u>42</u>-<u>86</u>**	4	7	4	6	6	8	6	41

The DNA sequences for 7 genes obtained from the Salmonella Genome Databases [22] and EnteroBase [23] were compared to determine different numbers of bases.

Allele types that differ from ST96 are underlined and in bold.

Table S5. *Salmonella enterica* serovar Schwarzengrund isolates obtained from chicken meat from various production regions.

District	No. of isolates	
	2008	2015-2019
West Japan	37	10
East Japan		49
Domestic [a]		28
Total	37	87

(a) The product district could not be identified.

Table S6. PCR conditions for detection of antimicrobial resistance genes and class 1 intergron.

Targeted gene	F/R*	Primer sequences 5' - 3'	Expected products size	Final primer conc.	Annealing Temp.	Positive control	Reference or Accession No.
Multiplex PCR 1							
aadA	F	GTG GAT GGC GGC CTG AAG CC	525 bp	0.1 µM	63℃	*S* . Schwarzengrund Sal_15	39
	R	AAT GCC CAG TCG GCA GCG				(in this study)	
strA/strB	F	ATG GTG GAC CCT AAA ACT CT	893 bp	0.4 µM		*S* . Schwarzengrund Sal_264	
	R	CGT CTA GGA TCG AGA CAA AG				(in this study)	
Multiplex PCR 2							
aphA1	F	ATG GGC TCG CGA TAA TGT C	634 bp	0.4 µM		*S* . Schwarzengrund Sal_15	39
	R	CTC ACC GAG GCA GTT CCA T				(in this study)	
aphA2	F	GAT TGA ACA AGA TGG ATT GC	347 bp	0.1 µM		*S* . Typhimurium Sal_243	
	R	CCA TGA TGG ATA CTT TCT CG				(unpublished)	
Multiplex PCR 3							
tetA	F	GGC GGT CTT CTT CAT CAT GC	502 bp	0.1 µM	63℃	*S* . Schwarzengrund Sal_17	39
	R	CGG CAG GCA GAG CAA GTA GA				(in this study)	
tetB	F	CGC CCA GTG CTG TTG TTG TC	173 bp	0.2 µM		*Escherichia coli* tori13	
	R	CGC GTT GAG AAG CTG AGG TG				(unpublished)	
Multiplex PCR 4							
sul1	F	CGG CGT GGG CTA CCT GAA CG	433 bp	0.2 µM	66℃	*S* . Schwarzengrund Sal_167	39
	R	GCC GAT CGC GTG AAG TTC CG				(in this study)	
sul2	F	CGG CAT CGT CAA CAT AAC CT	721 bp	0.3 µM		*S* . Schwarzengrund Sal_291	
	R	TGT GCG GAT GAA GTC AGC TC				(in this study)	

PCR 1							
dfrA14	F	ATR GCT GCG AAA GCG AAA AA	455 bp	0.5 µM	47℃	*S* . Schwarzengrund Sal_167	DQ388123, NG_047699, MT513102, CP062226
	R	CCC TTT TTC CAA ATT TGA TAG C				(in this study)	
PCR 2							
aac (*6'*)-*Iaa*	F	TGG CAT CAT TTA TTG CGA TG	237 bp	0.5 µM	57℃	*S* . Schwarzengrund Sal_167	CP053865
	R	TTG TAT TTT CCG GCG AGG TA				(in this study)	
PCR 3							
intI1	F	GCC TTG CTG TTC TTC TAC GG	558 bp	0.25 µM	55℃	*S* . Schwarzengrund Sal_G1	40
	R	GAT GCC TGC TTG TTC TAC GG				(in this study)	
PCR 4							
Variable region of class 1 integron							
	5' -CS	GGC ATC CAA GCA GCA AG	variable [(a)]	0.25 µM	55℃	*S* . Schwarzengrund Sal_G1	40
	3' -CS	AAG CAG ACT TGA CCT GA				(in this study)	

(a) variable size depending on the inserted gene(s).

*F, Forward; R, Reverse

Table S7. PCR primers used in the amplification and DNA sequencing for multilocus sequence typing.

Targeted gene	F/R*	Primer sequences 5' - 3'	Expected products size	Objective for each primer
thrA	F	GTC ACG GTG ATC GAT CCG GT	852 bp	PCR and DNA sequencing
	R	CAC GAT ATT GAT ATT AGC CCG		PCR and DNA sequencing
purE	F	ATG TCT TCC CGC AAT AAT CC	510 bp	PCR and DNA sequencing
	R	TCA TAG CGT CCC CCG CGG ATC		PCR and DNA sequencing
	F	CGC ATT ATT CCG GCG CGT GT		DNA Sequencing
sucA	F	AGC ACC GAA GAG AAA CGC TG	643 bp	PCR and DNA sequencing
	R	GGT TGT TGA TAA CGA TAC GTA C		PCR and DNA sequencing
hisD	F	GAA ACG TTC CAT TCC GCG CAG AC	894 bp	PCR and DNA sequencing
	R	CTG AAC GGT CAT CCG TTT CTG		PCR and DNA sequencing
	F	GTC GGT CTG TAT ATT CCC GG		DNA Sequencing
aroC	F	CCT GGC ACC TCG CGC TAT AC	826 bp	PCR and DNA sequencing
	R	CCA CAC ACG GAT CGT GGC G		PCR and DNA sequencing
hemD	F	ATG AGT ATT CTG ATC ACC CG	666 bp	PCR and DNA sequencing
	R	ATC AGC GAC CTT AAT ATC TTG CCA		PCR and DNA sequencing
	F	GTG GCC TGG AGT TTT CCA CT		DNA Sequencing
	R	GAC CAA TAG CCG ACA GCG TAG		DNA Sequencing
dnaN	F	ATG AAA TTT ACC GTT GAA CGT GA	833 bp	PCR and DNA sequencing
	R	AAT TTC TCA TTC GAG AGG ATT GC		PCR and DNA sequencing
	R	CCA TCC ACC AGC TTC GAG GT		DNA Sequencing

*F, Forward; R, Reverse

All primers were followed to the description in previous report [41].
